# Reassembly of Human Kidney Organoids after Dissociation

**DOI:** 10.1159/nef/adtag003

**Published:** 2026-09-09

**Authors:** Suzanne de Vetten, Annelot Klaassen, Dominique Y. Veldhoen, Thierry P.P. van den Bosch, Sander S. Korevaar, Ingrid M. Garrelds, Jan A.H. Danser, Jeroen van de Peppel, Sebastiaan Heidt, Martin J. Hoogduijn

**Affiliations:** a Division of Nephrology and Transplantation, Department of Internal Medicine, Erasmus MC Transplant Institute, Erasmus MC, Erasmus University Medical Center, Rotterdam, The Netherlands; b Department of Pathology and Clinical Bioinformatics, Erasmus MC, Erasmus University Medical Center, Rotterdam, The Netherlands; c Division of Pharmacology and Vascular Medicine, Department of Internal Medicine, Erasmus MC, University Medical Center, Rotterdam, The Netherlands; d Department of Internal Medicine, Erasmus MC, Erasmus University Medical Center, Rotterdam, The Netherlands

**Keywords:** Kidney organoids, Dissociation, Reassembly, Renin, Transfection

## Abstract

**Introduction:**

Human-induced pluripotent stem cell (iPSC)-derived kidney organoids have emerged as a valuable model for studying kidney development and disease. Phenotypically, these organoids resemble kidneys of gestational week 12. Embryonic kidneys have the capacity to reassemble after dissociation, which offers the possibility to study the effect of introducing additional cell types or gene expression modification through transfection of dissociated organoids before reassembly. We examined whether human iPSC-derived kidney organoids share the property of embryonic kidneys to reassemble after dissociation. This would open new research avenues with human kidney organoids.

**Methods:**

Human iPSC-derived kidney organoids were dissociated into single cells by TrypLE Select and reaggregated by centrifugation. Organoids were collected 4 and 7 days after reaggregation, and immunostaining was performed to assess structural organization. Restoration of endocrine activity was assessed by measurement of renin production. As a proof of principle, dissociated organoids were transfected with a GFP-expressing plasmid, and expression was monitored in reaggregated organoids.

**Results:**

Kidney organoids displayed the capacity to reassemble proximal and distal tubular structures, demonstrated by reappearance of villin-1^+^ and E-cadherin^+^ structures. Podocalyxin glomerular structures reappeared sparsely after dissociation. Reassembled organoids responded to forskolin with renin secretion, albeit at a lower level than non-dissociated organoids. Dissociated organoids that were transfected with a GFP plasmid at the cell suspension stage showed robust GFP expression after reassembly.

**Conclusion:**

Human kidney organoids partially reassemble after dissociation. Dissociation and reassembly of kidney organoids offers a novel tool for kidney organoid research that allows the introduction of exogenous cell types or transfection of kidney organoid cells.

## Introduction

Human-induced pluripotent stem cell (iPSC)-derived kidney organoids are a valuable in vitro research model for organogenesis and disease development studies and drug testing assays [1–3]. Kidney organoids are 3D structures that show structural organization and transcriptional profiles similar to kidneys of gestational week 12 [4, 5]. Over 20 different cell types are present in kidney organoids, which form glomerular and tubular-like structures and include a diversity of stromal cell types. In addition, kidney organoids contain specialized cell types that exhibit endocrine activity, such as renin and EPO production capacity [6, 7].

A limitation of the kidney organoid model is that its dense 3D architecture, combined with the absence of a perfusable endothelial network, restricts access to the organoid core. This limited accessibility makes it challenging to introduce exogenous cell types or to efficiently transfect organoid cells. Interestingly, mouse and human embryonic kidneys have the capacity to reassemble into organotypic kidney structures after dissociation into single-cell suspension [8, 9]. In addition, it has been shown that single-cell suspensions of embryonic kidney cells administered under the kidney capsule of adult rats generate vascularized nephrons [10]. These experiments raise the question of whether human iPSC-derived kidney organoids have a similar capacity to reassemble after dissociation. This would offer novel opportunities for kidney organoid research.

In the present study, we examined whether human iPSC-derived kidney organoids have the ability to reaggregate and structurally reorganize after dissociation. In addition, tubular transporter expression and renin production capacity of the reassociated organoids were investigated. Finally, we explored a functional application for this procedure.

## Methods

### iPSC Derivation and Culture

Human erythroid progenitors from three healthy donors were reprogrammed to iPSCs as described earlier [11] after obtaining written informed consent of the donors and according to protocols approved by the Medical Ethics Committee of the Erasmus University Medical Center (MEC-2017-248) (Table 1). iPSCs were cultured and expanded on plates coated with Geltrex (Gibco, USA) in StemFlex medium (Gibco). The cells were cultured for a minimum of 2 weeks prior to the start of organoid differentiation. Cells were passaged upon reaching 70–80% confluency using ReLeSR (STEMCELL Technologies, Canada). The iPSC lines used are registered in the Human Pluripotent Stem Cell Registry (EMCi227-A, EMCi169-B, EMCi353-A).

**Table 1.** Details of the used iPSC lines

​	Tissue of origin	Gender	Age	Reprogramming method
iPSC 1	Erythroid progenitors	Male	26	Sendai virus
iPSC 2	Erythroid progenitors	Male	∼17	Sendai virus
iPSC 3	Erythroid progenitors	Male	40	Sendai virus

### Kidney Organoid Differentiation

Kidney organoids were differentiated from iPSCs as previously described [5, 12, 13]. In brief, at day −1, iPSCs were detached with TrypLE Select (Thermo Fisher Scientific, USA) and seeded at 3×10^4^ cells/cm^2^ in StemFlex medium supplemented with RevitaCell Supplement (Thermo Fisher Scientific). The following day, differentiation was initiated by switching to Advanced RPMI 1640 medium (Gibco, USA) with 8 µm CHIR99021 (Bio-Techne, USA). Medium was refreshed daily. After 3 days, medium was replaced with Advanced RPMI 1640 supplemented with 10 ng/mL Activin A (R&D Systems, USA), 200 ng/mL Fibroblast growth factor 9 (FGF9) (PeproTech, USA) and 1 µg/mL Heparin (Sigma, USA). On day 4, cells were detached using TrypLE Select, washed and resuspended in Advanced RPMI 1640 supplemented with 3 µm CHIR99021, 200 ng/mL FGF9 and 1 µg/mL Heparin. 5×10^5^ cells/well were seeded in a V-bottom 96-well plate and centrifuged at 300 g for 3 min. The cell aggregates were incubated at 37°C, 5% CO_2_ and 100% humidity for 48 h. Next, the cell aggregates were placed on 6-well plate Transwell membranes (cellQART, Germany) and cultured at the air-liquid interface with 1.2 mL Advanced RPMI 1640 supplemented with 3 µm CHIR, 200 ng/mL FGF9, and 1 µg/mL Heparin in the lower well. After 24 h, the medium was replaced with Advanced RPMI 1640 supplemented with 200 ng/mL FGF9 and 1 µg/mL Heparin and refreshed every 2 days for 4 days. On day 11, the medium was replaced with Advanced RPMI 1640 without growth factors and refreshed every other day until collection.

### Dissociation and Reaggregation of Organoids

On day 17 of differentiation, organoids were collected and enzymatically dissociated into a single-cell suspension using TrypLE Select for 8 min. Cell suspensions were filtered through a 40-µm cell strainer and centrifuged at 300 g for 4 min. Cells were then washed and resuspended in Advanced RPMI 1640 medium supplemented with 10 µm of the Rho-kinase inhibitor Y-27632 (Bio-Techne). To reaggregate kidney organoids, 5×10^5^ cells were added per well of a 96-well plate, which was centrifuged at 300 g for 3 min and incubated at 37°C and 5% CO_2_. The next day (D17 + 1), cell aggregates were transferred to the Transwell culture system described above. Organoids were collected on day 4 (D17 + 4) and day 7 (D17 + 7) after reaggregation. Undissociated control organoids were collected on day 20.

### Transfection of Dissociated Organoids

Dissociated organoids were transfected with pmaxGFP DNA plasmid using the P3 Primary Cell 4D-Nucleofector^®^ X Kit (Lonza). The CA-137 program was used for transfection. After transfection, cells were reaggregated as described above. Organoids were kept in culture for 7 days. GFP expression was assessed at 4.5 h, 24 h, 3 days, and 7 days post-transfection by detecting the green fluorescent signal through fluorescence microscopy.

### Immunohistochemical and Immunofluorescence Staining

Organoids were harvested and fixed in 4% paraformaldehyde, embedded in paraffin, and cut in 4-µm slices. Deparaffinization and antigen retrieval were performed by heating in Tris/EDTA buffer (ab93684, Abcam, UK). Antigen blocking was performed with 5% normal goat serum in 0.1% PBS-Tween. For immunohistochemical (IHC), E-cadherin (Mouse, clone 36, Becton Dickinson, USA, 1:100), Villin-1 (Rabbit, clone EPR3491, Abcam, 1:800), and PODXL (Rabbit, clone EPR9518, Abcam, 1:1000) were used. Slides were incubated with HRP polymer antibodies (Bio-Techne [Rabbit] and Abcam [Mouse]), and DAB staining and hematoxylin counterstaining were performed. For immunofluorescence (IF), LTL (Fluorescein labeled, Vector laboratories, UK, 1:200), E-cadherin (Mouse, clone 36, Becton Dickinson, USA, 1:200), and PODXL (Rabbit, clone EPR9518, Abcam, 1:100) were used. Appropriate polyclonal Alexa Fluor-conjugated secondary antibodies (Abcam) were used, and DAPI was used as a nuclear counterstain.

Staining of Na^+^K^+^Cl^−^ cotransporter 2 (NKCC2) and co-staining of renin and connexin40 (C×40) was performed by automated multiplex IF using the Ventana Benchmark Discovery (Ventana Medical Systems Inc., AZ, USA). In brief, following deparaffinization and heat-induced antigen retrieval with CC1 (#950-500, Ventana) for 32 min the tissue samples were incubated with NKCC2 (Rabbit, StressMarq, Polyclonal, 1:400) or Renin (Rabbit, Abcam, clone EPR20693, 1:2500) and incubated for 32 min at 37°C followed by OmniMAP-rabbit HRP (#760-4311, Ventana) and detection with Red610 (#760-245, Ventana) for 8 min. An antibody denaturation step was performed with CC2 (#950-123, Ventana) at 100°C for 20 min. Secondly, incubation with anti-Connexin40 (Rabbit, Invitrogen, Polyclonal, 1:100) was performed for 32 min at 37°C, followed by OmniMAP-mouse HRP (#760-4311, Ventana) and detection with FAM (#760-243, Ventana) for 8 min. Finally, slides were washed in phosphate-buffered saline and mounted with VectaShield containing 4′,6-diamidino-2-phenylindole (Vector Laboratories). Slides were imaged with Axioscan 7 (Zeiss, Germany).

### Quantification of IHC Staining

Quantification of villin-1, podocalyxin (PODXL), and E-cadherin IHC staining was performed using QuPath software (version 0.5.1). First, the total organoid area was manually annotated. Subsequently, a threshold was set to distinguish the positively stained areas from unstained areas. Finally, the positively stained surface area was determined by the software.

### Renin Measurement

To measure renin activity, D17 + 7 organoids were treated with 10 µm forskolin for 24 h. Subsequently, conditioned medium was collected for renin activity assay. Renin levels were measured using the DSL IRMA Active Renin kit (Beckman Coulter Inc., Brea, CA). The detection limit of the IRMA kit was 0.81 pg/mL, with a standard curve ranging from 5 to 500 pg/mL.

## Results

### Reassembled Organoids Contain Reorganized Tubular Structures

To investigate whether dissociated kidney organoids were capable of reorganization, organoids were harvested for analysis 4 and 7 days after reaggregation (Fig. 1a). Reassembled organoids were smaller than day 20 control organoids. IHC analysis demonstrated that villin-1^+^ proximal tubular cells and E-cadherin^+^ distal tubular cells were reorganized in tubular-like structures on day 7 after dissociation (Fig. 1b). There was some variation in the kinetics of reassembly, as in two iPSC lines, reorganized tubular structures were already visible after 4 days, while in another line this took 7 days. The tubular-like structures were denser, and more appeared to be more connected in reassembled organoids than in control organoids. In contrast to the tubular cells, PODXL^+^ glomerular cells were strongly reduced in number and largely lost their organization in glomerular-like structures both at day 4 and day 7. Quantification of the IHC staining demonstrated that the area of PODXL^+^ structures was significantly reduced in reassociated organoids at day 4 and day 7, while villin-1^+^ and E-cadherin^+^ tubular structure area did not change (Fig. 1c). To examine the nephron structures in reassembled organoids in more detail, we compared triple IF staining for glomerular (PODXL), proximal tubular (LTL), and distal tubular (E-cadherin) cells in control and D17 + 7 organoids. The images confirmed the IHC findings that tubular structures reappear after dissociation and that glomerular structures are largely lost (Fig. 1d).

**Fig. 1.**
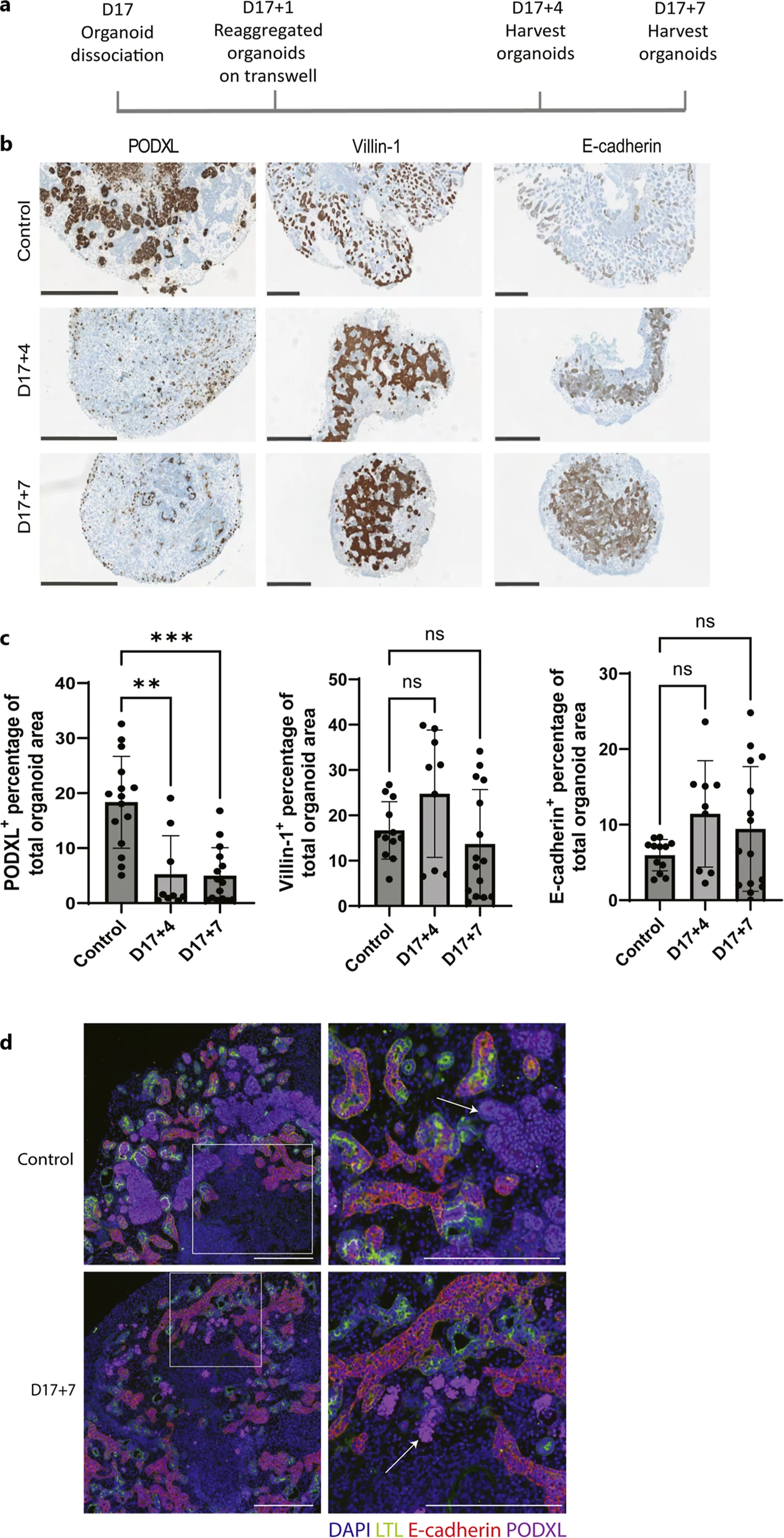
Human kidney organoids partially reassemble after dissociation. **a** Timeline of organoid dissociation, culture and harvest. **b** IHC staining of PODXL, villin-1 and E-cadherin in control and dissociated organoids at days 4 and 7 after reaggregation. The scale bar represents 500 µm. **c** Quantification of positive staining area for PODXL, villin-1 and E-cadherin. **d** Triple IF staining for PODXL (purple), LTL (green) and E-cadherin (red) in control and dissociated organoids at day 7 after reaggregation. Arrows point to glomerular structures in control organoids and to incompletely restructured glomerular structures in reassociated organoids. The white boxes indicate the zoomed-in area shown on the right. The bars represent 250 µm. The experiments were performed three times with different iPSC lines; images of a representative experiment are shown.

### Reassembled Organoids Regain Renin Secretion Capacity and Maintain Tubular Transporter Expression

Kidney organoids contain a variety of stromal cells that contribute to extracellular matrix formation and endocrine activity, including a subset of renin-producing cells. To investigate whether renin-producing cells maintain their function in reassembled organoids, kidney organoids were stimulated with forskolin, a known inducer of renin production in kidney organoids [6], and stained for renin and C×40, a marker for renin-producing cells and endothelial cells of the kidney [14]. Renin was absent in organoids that were not stimulated with forskolin. Upon forskolin stimulation, renin was visible in a subset of C×40^+^ cells in both control and reassembled organoids (Fig. 2a).

**Fig. 2.**
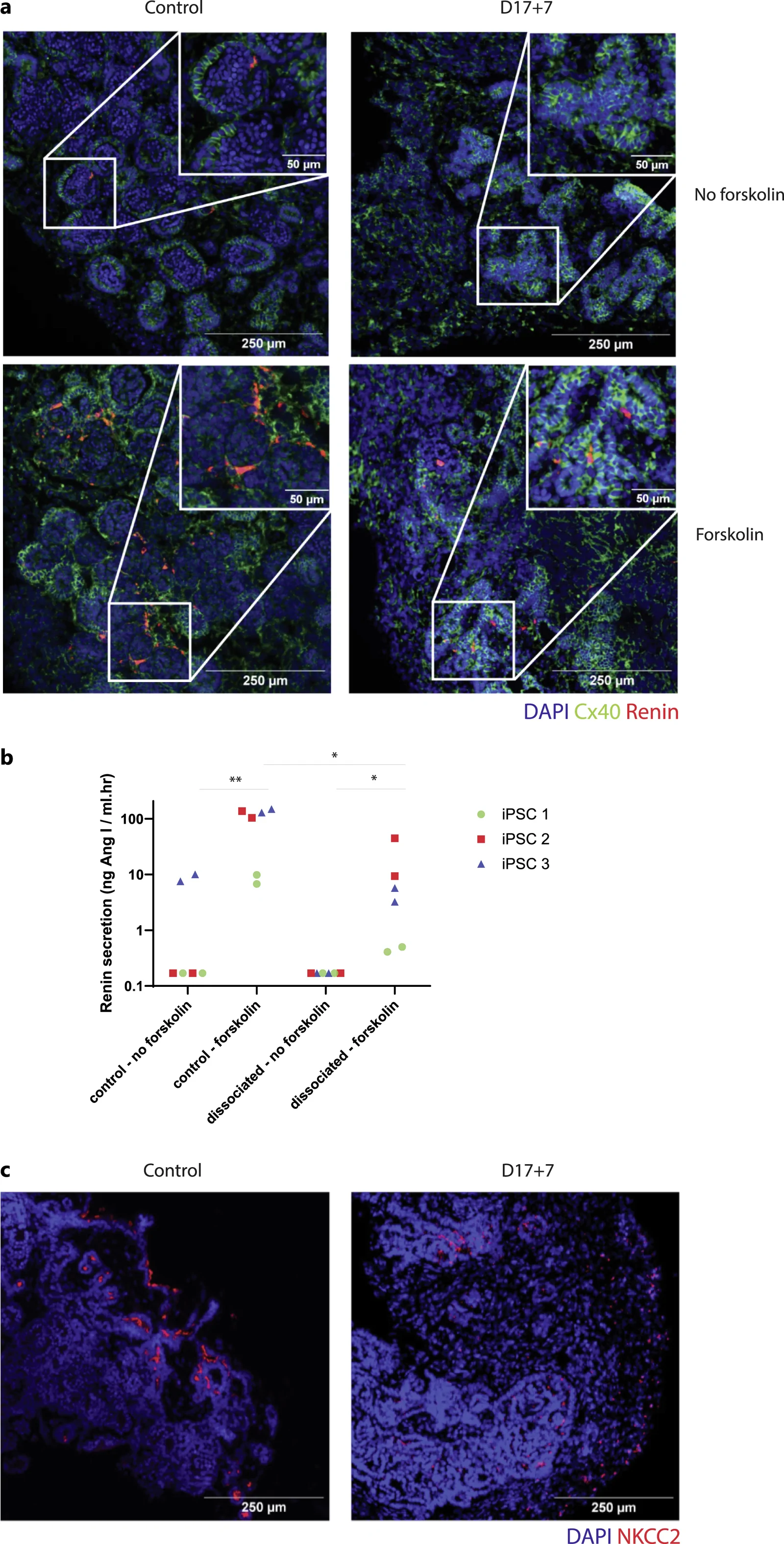
Reassembled organoids regain renin secretion capacity after forskolin treatment and maintain tubular transporter expression. **a** IF staining of renin (red) in control and reassembled organoids with and without forskolin treatment. Green color marks C×40. **b** Renin secretion of control and reassembled kidney organoids from iPSC lines 1, 2 and 3 before and after forskolin treatment, represented as ng angiotensin I/mL.hr. **c** NKCC2 IF (red) staining of control and reassembled organoids at day 7.

Both forskolin-stimulated control and dissociated organoids were capable of releasing renin in the cell culture medium (Fig. 2b). Renin release by dissociated organoids did not reach the levels of those of control organoids (*p* = 0.017). To examine whether reassociated tubular structures maintained expression of tubular transporters, we stained for NKCC2 at day 7 after reaggregation. NKCC2 remained expressed in reassociated tubular structures (Fig. 2c).

### Transfection of Dissociated Organoids

To demonstrate one proof-of-principle application for the dissociation and subsequent reaggregation of kidney organoids, we transiently transfected dissociated organoids with a DNA vector carrying GFP (Fig. 3a). GFP was abundantly expressed in reaggregated organoids from 4.5 h up to 3 days after transfection and faded out at day 7 (Fig. 3b). These results demonstrate that it is possible to efficiently transfect kidney organoids after dissociation. IHC analysis demonstrated that villin-1^+^ and E-cadherin^+^ structures were present in transfected and reassociated organoids, while PODXL^+^ cells lost their structural organization (Fig. 3c), similar to non-transfected controls (Fig. 1).

**Fig. 3.**
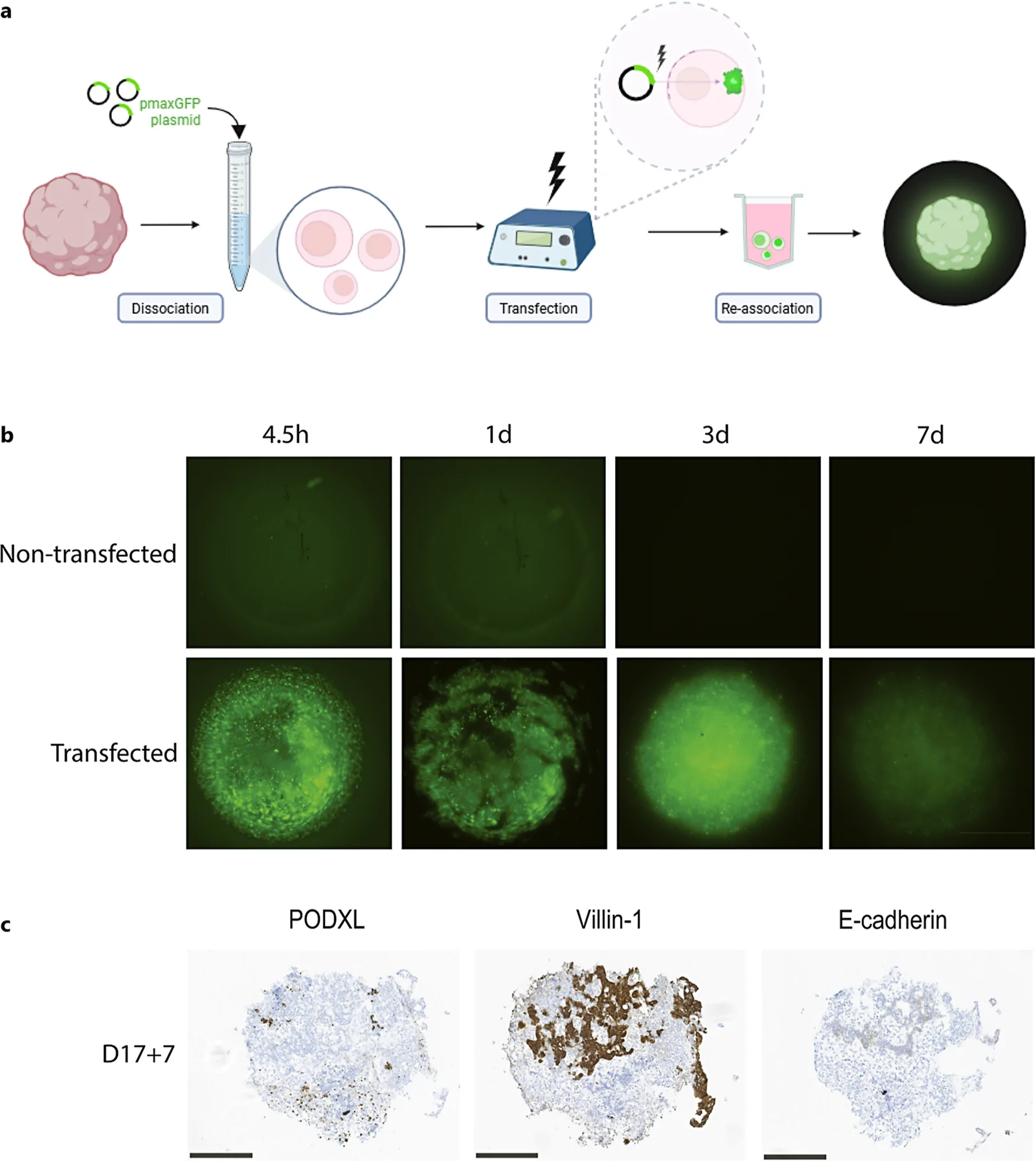
Transfection of kidney organoids after dissociation. **a** Experimental procedure of transfection of kidney organoids with a GFP-DNA vector after dissociation and subsequent reaggregation. **b** GFP expression in transfected and non-transfected organoids at 4.5 h, 24 h, 3 days, and 7 days after transfection. The scale bar represents 250 µm. **c** PODXL, villin-1, and E-cadherin IHC staining on transfected and reassembled organoids. The scale bar represents 250 µm.

## Discussion

We demonstrated that iPSC-derived kidney organoids have the capacity to reassemble organotypic kidney structures after dissociation. Proximal and distal tubular structures that reappeared 4–7 days after reaggregation displayed more elongated and interconnected structures than in control organoids. Glomerular structures did not reorganize after dissociation, potentially reflecting the limited regenerative capacity of podocytes [15]. Dissociation and reaggregation of kidney organoids may therefore be a model to study the mechanisms of the poor regenerative capacity of podocytes. We furthermore showed that forskolin treatment of reassembled kidney organoids induced the secretion of renin, albeit not at the level before dissociation, which indicates that the stromal cell fraction in reassembled organoids partially regains one of its multitude of functions. It is interesting that kidney-derived renin-producing juxtaglomerular cells lose their renin secretion capacity in culture [16], suggesting that the organoid microenvironment supports renin-producing cells.

The partial restoration of cellular organization in kidney organoids after dissociation aligns with the reorganization capacity of embryonic kidneys [8, 9]. The partial reassembly of kidney organoids, in contrast to the complete regain of kidney morphogenesis of dissociated embryonic kidneys, can potentially be explained by the lack of sufficiently plastic progenitor cells in kidney organoids. Unbekandt et al. demonstrated the complete reorganization of dissociated kidneys derived from E11.5 mouse embryos [8]. This embryonic state corresponds to approximately 5–6 weeks of human development. Mature human kidney organoids resemble late first to early second trimester human fetal kidneys (gestational week 10–12) [2, 4, 5]. It is therefore possible that the kidney organoids that were dissociated in our study were too mature to fully reassemble and that reassembly of more immature organoids would allow glomerular reorganization and full restoration of renin-producing cells. It is, however, difficult to compare embryonic stages between human and mouse, as human and mouse kidney development differ in timing [17]. Despite its limitations, the partial reassembly of human kidney organoids represents a useful model to study human kidney development in a modifiable in vitro system and to examine the differential regenerative properties of different kidney cell types.

The dissociation and reassembly of kidney organoids allows experimental manipulation of kidney organoids that would otherwise not be possible. For instance, gene expression modification via viral transfection or CRISPR-Cas9 is not possible in fully grown kidney organoids, as viruses and other carriers poorly penetrate through the multiple cell layers of the organoids. Therefore, these treatments can be applied only to iPSC cell suspensions at the start of the differentiation protocol or when cells are brought into suspension during the transition from 2D to 3D culture on day four of the organoid differentiation procedure. Targeting more mature kidney-like cell types in organoids is thus not possible. Early treatment may furthermore interfere with the differentiation process of kidney organoids unwantedly. The ability to dissociate and reassemble kidney organoids at the end of the differentiation protocol allows transfection in mature organoids. This offers the opportunity to study the role of genes of interest in kidney disease development or response of kidney cells to drugs. In addition, dissociation and reassembly allow the introduction of novel cell types in kidney organoids, such as, for instance, endothelial cells to study reendothelialization processes.

The kidney contains multiple populations of stromal cells with diverse functions located in the interstitial space. In our study, we examined the restoration of just one function of a subpopulation of stromal cells through measurement of renin production. The ability of dissociated organoids to produce renin after forskolin treatment implies that stromal cells in reassembled organoids are still functional. In the kidney, renin-producing cells are marked by C×40, which is also expressed on endothelial cells. C×40 acts as an intercellular signaling protein [14]. In both control and reassembled kidney organoids, renin-producing cells were in proximity to C×40^+^ cells, suggesting a connection between renin-producing cells and endothelial cells was restored in reassembled organoids. It has been demonstrated that renin-producing cells emerge in kidney organoids after enhancement of the endothelial niche through genetic engineering [18]. Dissociation of organoids and supplementation with endothelial cells offers a tool to further study the mechanisms of the interaction between endothelial cells and renin-producing cells.

In addition to the ability of reassembled kidney organoids to produce renin, we demonstrated the presence of the tubular transporter NKCC2 on reassembled organoids. The expression of this transporter on tubular structures on kidney organoids indicates that at least part of the apparatus for tubular transport is present on kidney organoids, and its continued expression demonstrates that this is also the case for reassembled organoids. To assess the broad spectrum of cellular function after reassembly of kidney organoids, additional measurements are required, such as, for instance, the ability of pericytes to secrete EPO in response to hypoxia or the deposition of extracellular matrix proteins by myofibroblasts in response to injury to model disease processes.

A limitation of the current reassembly protocol is that kidney organoid reassembly remains incomplete. The reduction of PODXL-positive cells in the reassembled organoids compared to control organoids, as well as the significant decrease in renin production after reassembly, demonstrate that the organoids do not return to exactly the same state as before dissociation. In addition, the prevalence of villin + proximal tubular cells in reassembled organoids increases. Further optimization of the dissociation and reassembly protocol may enhance the ability of organoids to return to their original state after dissociation, which would increase the translational relevance of reassembled organoids.

In conclusion, we demonstrated that kidney organoids partially reassemble after dissociation and showed a proof-of-principle application by transfection of dissociated organoids with a GFP-DNA vector. Other potential applications include the introduction of exogenous cells in kidney organoids and studies of the regenerative potential of different compartments of the human kidney. The dissociation-reaggregation protocol is therefore a contribution to the toolbox of kidney organoid research.

## Statement of Ethics

iPSCs were generated from human donors as part of the project ‘Creation of disease model systems to understand and correct genetic diseases through gene or other therapy using iPSC derived from somatic cells: iPSC protocol Rotterdam approved by the Erasmus MC Medical Ethics Committee under approval no.: MEC-2017-248, which was published on March 29th, 2018, and last updated on April 15th, 2024.

## Conflict of Interest Statement

There are no conflicts of interest to report.

## Funding Sources

The authors declare that no funding was obtained or used for this study.

## Author Contributions

S.V.: conducted experiments and wrote manuscript; D.V., A.K., S.K., and I.G.: conducted experiments; T.vdB.: conducted experiments and proofread manuscript; A.D.: proofread manuscript; J.P.: technical assistance and proofread manuscript; S.H.: conceptual design and proofread manuscript; M.H.: conceptual design and wrote manuscript.

## Data Availability

All data are included in the article and its Supplementary Information files or are available from the corresponding authors upon reasonable request.
